# Dengue and Zika Virus Domain III-Flagellin Fusion and Glycan-Masking E Antigen for Prime-Boost Immunization

**DOI:** 10.7150/thno.35919

**Published:** 2019-07-09

**Authors:** Hsiao-Han Lin, Shao-Ping Yang, Meng-Ju Tsai, Guan-Cheng Lin, Han-Chung Wu, Suh-Chin Wu

**Affiliations:** 1 Institute of Biotechnology, National Tsing Hua University, Hsinchu 30013, Taiwan; 2 Institute of Cellular and Organismic Biology, Academia Sinica, Taipei 11529, Taiwan; 3 Department of Medical Science, National Tsing Hua University, Hsinchu 30013, Taiwan

**Keywords:** flagellin-domain III fusion, dengue and Zika viruses, neutralizing antibodies, cross-reactive antibodies, prime-boost vaccines

## Abstract

The viral E proteins of dengue virus (DENV) and Zika virus (ZIKV) are the major viral proteins involved in receptor binding and fusion, and for the induction of protective antibodies against viral infections. DIII of the E proteins is an independent domain and stretches out on the virion surface that can elicit type-specific neutralizing antibodies. For recombinant DIII vaccine development, prime-boost immunizations can provide an advantage of eliciting more type-specific neutralizing antibodies by recalling DIII antigens after DIII booster to improve protection.

**Methods**: The DIII of the E genes of DENV and ZIKV were fused with bacterial *fliC* gene for the expression of flagellin-DIII (FliC-DIII) fusion proteins. Prime-boost immunization strategies by the second-dose booster of four DENV serotype or ZIKV FliC-DIII fusion proteins were used to investigate the induction of neutralizing antibodies and protection against viral infections. Cross-reactive non-neutralizing antibodies in each group of antisera were also examined using *in vitro* antibody-dependent enhancement (ADE) assay. A series of glycan-masking E antigens were finally constructed for prime-boost immunizations to abolish the elicitation of cross-reactive non-neutralizing antibodies for ADE activity.

**Results**: We showed that inclusion of a bivalent live-attenuated vaccine with a FliC-DIII booster is superior in eliciting neutralization titers and protection *in vivo* against all four-serotype DENVs. We also demonstrated that recombinant adenovirus vectors encoding four-serotype DENV prMEs with a FliC-DIII prime-boost scheme is capable of eliciting good antibody responses. In contract, recombinant adenovirus vector of ZIKV prME gene priming, followed by ZIKV FliC-DIII booster did not improve vaccine efficacy. The glycan-masking mutation on the ZIKV E protein ij loop (E-248NHT), but not on DENV2 E protein ij loop (E-242NHT), resulted in abolishing the elicitation of cross-reactive antibodies for DENV and ZIKV infection enhancements.

**Conclusions**: Our findings can provide useful information for designing novel immunogens and vaccination strategies in an attempt to develop a safe and efficacious DENV or ZIKV vaccine.

## Introduction

Dengue virus (DENV) and Zika virus (ZIKV) are small-enveloped positive-strand RNA viruses belonging to the *Flavivirus* genus of the *Flaviviridae* family 1. DENV is comprised of four serotypes (DENV1, DENV2, DENV3, and DENV4), and the primary infection of one of the DENV serotypes causes mild and self-limited dengue fever; however, secondary infection of the other DENV serotypes may cause life-threatening conditions, including dengue hemorrhagic fever and dengue shock syndrome 1. It has been estimated that approximately 390 million DENV infections occur annually, of which 96 million are manifested as clinical diseases 2. The outbreaks of ZIKV infections in 2014 in Brazil have resulted in significantly increased cases of neonatal microcephaly, Guillain-Barre syndrome (GBS), and congenital abnormalities 3-7. The World Health Organization (WHO) thus declared the ZIKV infections of 2016 as a “Public Health Emergency of International Concern” 8.

The DENV and ZIKV genome encodes three structural genes (capsid C, membrane precursor prM, and envelope E) and seven non-structural (NS1, NS2A, NS2B, NS3, NS4A, NS4B, and NS5) genes, with untranslated region (UTR) genes flanking the 5′ and 3′ ends 1. E protein is the major viral protein involved in receptor binding and fusion, and is formed as a head-to-tail dimer on the surfaces of viral particles 9,10. E protein consists of three distinct domains: a central beta-barrel domain I (DI), an extended finger-like dimerization domain II (DII), and an immunoglobulin-like domain III (DIII) 9,10. DI and DII are discontinuous peptides connected by four peptide linkers, which form the DI/DII hinge. DI acts as a bridge between DII and DIII. The DI/DII hinge is important for turning over DII to expose the fusion loop (FL) during the fusion process. DIII is a relatively independent domain and stretches out on the virion surface.

Recombinant DENV DIII antigens have been reported to elicit type-specific neutralizing antibodies against all four DENV serotypes in mice and nonhuman primates 11-17. Recombinant ZIKV DIII antigens were also reported to induce neutralizing antibodies in mice 18-20. The heterologous prime-boost strategy using live-attenuated vaccines (LAVs) priming, followed by DENV DIII boosting was found to further augment the anti-DENV immune responses 21. Prime-boost immunizations with DIII boosting regimen can further recall DIII antigen to elicit long-lasting neutralizing antibodies following LAV infections in monkeys 21.

The sequence analysis of E protein previously demonstrated that DENV and ZIKV are more phylogenetically close to each other than other flaviviruses 22. It was initially proposed for use in DENV infections for disease-enhancing antibody- dependent enhancement (ADE) activity: the antibodies induced by primary DENV infections can enhance secondary infections through cross-reactive non-neutralizing antibodies by facilitating virus entry to FcγR-bearing myeloid cells 23. Therefore, the development of safe and effective vaccines is needed to abolish the elicitation of cross-reactive non- neutralizing antibodies between DENV and ZIKV 24-25. Epitope mapping studies have shown that most of these cross-reactive antibodies are induced by the DI/DII regions of E protein, particularly by the highly conserved FL of the DII region, which promotes DENV- or ZIKV-enhancing infections 25-29. Cross-reactive ADE activity has been found using human monoclonal antibodies isolated from patients, indicating that FL-directed non- or low- neutralizing monoclonal antibodies are cross-reactive among different DENV serotypes or between DENV and ZIKV 22,28,30-31. Previous studies have attempted to reduce the production of cross-reactive antibodies through antigen design, with five mutations (G106R, L107D, K310D, E311K, P364Q) in or near the FL for a DENV DNA vaccine 32,33 and four mutations in or near the DII/FL (T76R, Q77E, W101R, and L107R) for a ZIKV mRNA vaccine 34.

In this study, we evaluated the immune responses and cross-reactivity between DENV and ZIKV by refocusing on E protein DIII. The DIII of the E genes of DENV1-4 and ZIKV were fused with bacterial *fliC* gene for the expression of flagellin-DIII (FliC-DIII) fusion proteins. We applied a prime-boost strategy, using DENV LAVs for a bivalent priming or adenovirus vectors expressing DENV prME for a tetravalent priming, followed by FliC-DIIIs booster to induce immune responses against four serotypes of DENVs. In parallel, we constructed adenoviral vector expressing ZIKV prME for priming, followed by FliC-DIII booster immunization to elicit neutralizing antibodies against ZIKV infection. Finally, we designed a series of glycan-masking mutants on the ij loop of the DENV or ZIKV E protein for prime-boost immunizations and investigated antisera for cross-reactivity between DENV and ZIKV to abolish the ADE activity. These results may provide useful information for designing novel immunogens and vaccination strategies in an attempt to develop a safe and efficacious dengue or Zika virus vaccine.

## Results

### Expression and characterization of recombinant FliC-DIII proteins of four-serotype DENVs and ZIKV

**Figure 1:**
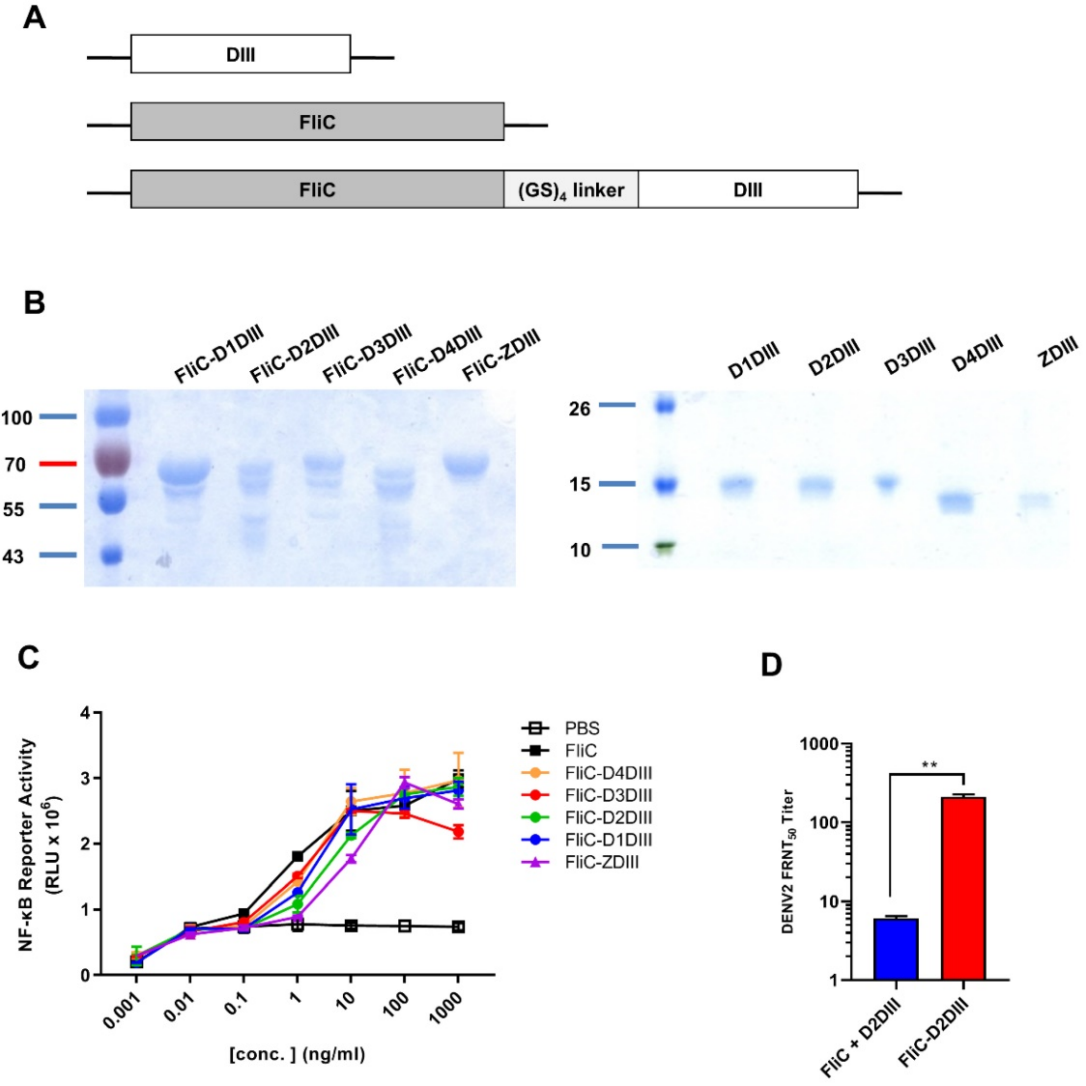
** Expression and characterization of recombinant DIII and FliC-DIII proteins. (A)** DIII, FliC, and recombinant protein FliC and DIII, which were linked by GS_4_ linker, were constructed into the pET-22b(+) vector with a C-terminal His-tag for protein expression proteins.** (B)** The DIII proteins (D1DIII, D2DIII, D3DIII, D4DIII, ZDIII) and the FliC-DIII fusion proteins (FliC-D1DIII, FliC-D2DIII, FliC-D3DIII, FliC-D4DIII, and FliC-ZDIII) were expressed by *E.coli* BL21 (DE3) and purified using nickel-chelated affinity chromatography. Purified proteins were verified by SDS-PAGE staining with Coomassie blue. **(C)** TLR5 signal functional assay was performed by co-culturing the10-fold serial dilutions of FliC or recombinant proteins with HEK 293A expressing hTLR5 receptor and NF-κB reporter vector. The cells were disrupted and treated with Neolite luciferase substrate. The TLR5 activity was measured by luciferase activity. **(D)** BALB/c mice were immunized two doses of 20 μg FliC-D2DIII or a mixture of 20 μg FliC and 20 μg D2DIII in a 3-week interval. Sera were collected 2 weeks after the second-dose immunizations and analyzed by FRNT assay. FRNT_50_ values were calculated by GraphPad Prism 6. (**, p <0.01).

The DIII cDNAs of the E genes of DENV1 (Hawaii), DENV2 (NGC), DENV3 (H87), DENV4 (2A), and ZIKV (PRVABC59) were constructed into the pET-22b(+) vector for the expression of DIII proteins (D1DIII, D2DIII, D3DIII, D4DIII, and ZDIII). These DIII cDNAs were also fused with the *fliC* gene from *Salmonella typhimurium* by a GS_4_ linker for the expression of FliC-DIII fusion proteins (FliC-D1DIII, FliC-D2DIII, FliC-D3DIII, FliC-D4DIII, and FliC- ZDIII), (Fig. 1A). These recombinant proteins were all obtained from *E. coli* and purified using nickel- chelated affinity chromatography. As shown in Fig. 1B, the purified proteins had a molecular weight around 64 kDa for the FliC-DIII fusion proteins and a molecular weight around 13 kDa for DIII proteins as shown in SDS-PAGE gels with Coomassie blue staining (Fig. 1B). We noted that the presence of secondary bands for these FliC-DIII proteins as shown in Fig. 1A, which is likely due to proteinase digestion as we and other reported for recombinant FliC expression 35-36. The soluble form of FliC protein may lose its tertiary structure and becomes sensitive to proteolytic degradation on the D0 domain of FliC molecules that contain D0, D1, D2, and D3 domains 37. These FliC-DIII fusion proteins were examined for their ability to trigger TLR-5 signaling pathways in HEK-293 cells. The results indicated that the purified recombinant FliC and all of the five FliC-DIII fusion proteins (FliC-D1DIII, FliC-D2DIII, FliC-D3DIII, FliC- D4DIII, and FliC-ZDIII) triggered TLR-5 signaling at a similar range in the luminescence values driven by the NF-κB reporter gene expression in HEK-293 cells (Fig. 1C). Immunizations with FliC-D2DIII compared to the mixture of FliC and D2DIII in BALB/c mice were conducted via two doses in a 3-week interval. Sera were collected at 2 weeks after the second dose of immunizations. The results indicated that the neutralizing antibody titer (FRNT50 value) for the FliC-D2DIII fusion protein immunized group was significantly higher than that of the immunized group for the mixture of FliC and D2DIII (Fig. 1D). Therefore, recombinant DIII proteins with flagellin fusion were used for the following prime-boost immunization studies.

### DENV LAV (2,4) priming, followed by FliC-DIII(1-4) booster immunizations in AG129 mice

**Figure 2:**
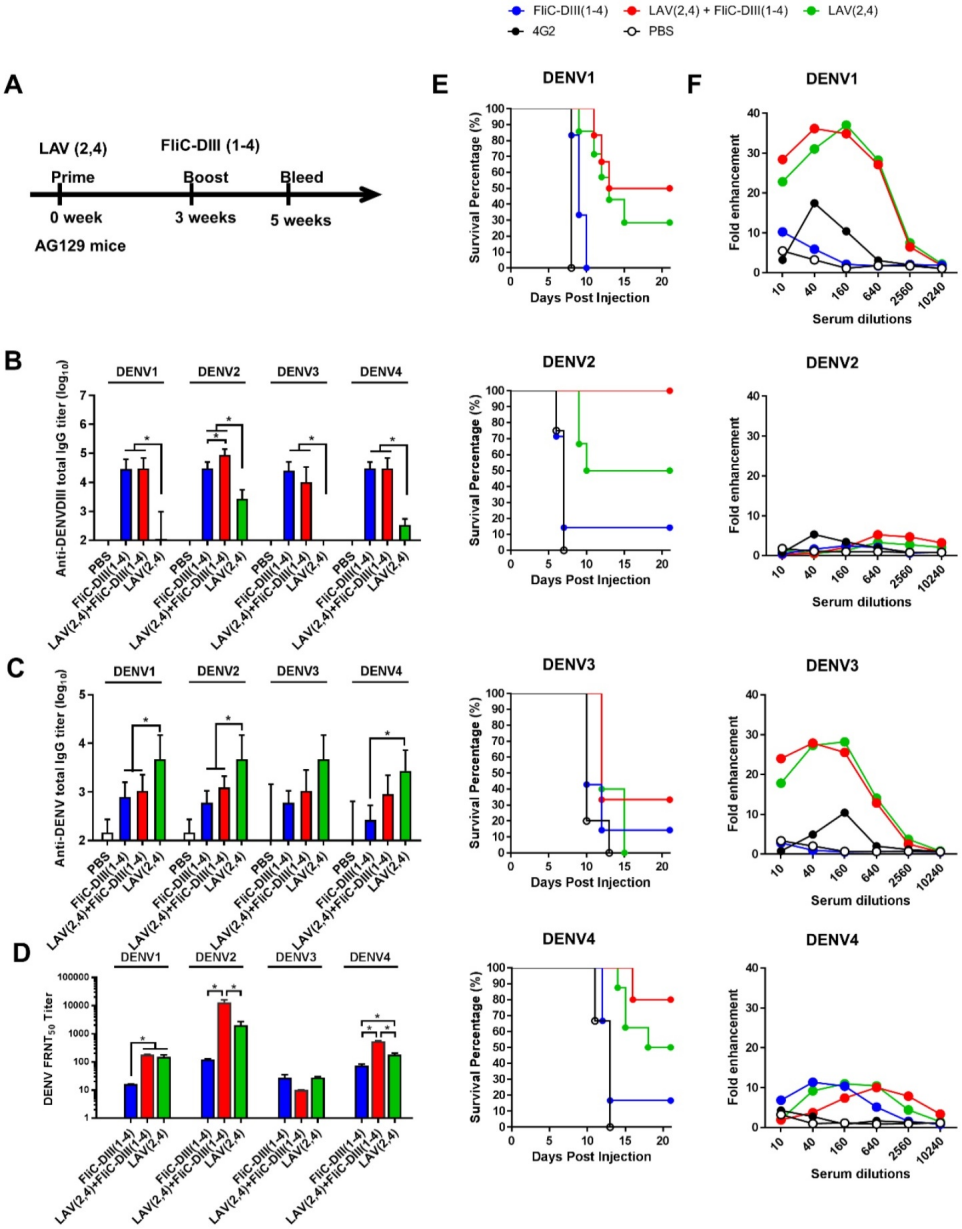
** Live-attenuated virus vaccine priming, followed by FliC-DIII booster immunizations in AG129 mice. (A)** Prime-boost immunization regimens were conducted using the NIH live-attenuated bivalent vaccine strains (DENV2/4Δ30 and DENV4Δ30, or LAV(2,4)) and tetravalent FliC-DIII(1-4) of DENV1-4 serotypes. Groups of AG129 mice were immunized using LAV(2,4) priming, followed by FliC-DIII(1-4) booster, or two doses of tetravalent FliC-DIII(1-4) or LAV(2,4). PBS-immunized group was the control. Sera were collected from the mice two weeks after the second dose immunization. **(B)** Serum IgG titers against four DENV serotypes were determined by ELISA coated with recombinant DIII proteins. **(C)** Serum IgG titers against four DENV serotypes were determined by ELISA coated with DENVs. **(D)** Neutralizing antibody titers against DENV1, DENV2, DENV3, or DENV4. **(E)** Antisera passive protection in newborn mice. Antisera from each immunized groups were pooled and mixed with DENVs, and then injected into to newborn 129 mice intracranially for measuring protection. The survival rates of mice were recorded daily. **(F)** ADE activity of DENV infection mediated by each group of antisera in K562 cells. The mAb 4G2 was the positive control. The fold enhancement of ADE activity was calculated by dividing the percentage of infected cells with antisera by the percentage of infected cells without antisera. Data were analyzed using one-way ANOVA. (*, p <0.05).

To investigate whether immunizations with the second dose of FliC-DIII booster can further enhance neutralizing antibody responses against the four DENV serotypes, prime-boost immunization regimens were conducted using the NIH live-attenuated bivalent vaccine strains (DENV2/4Δ30 and DENV4Δ30, or LAV(2,4)) and tetravalent FliC-DIII (1-4) of DENV1-4 serotypes (**Fig. 2A**). Type-I & II interferon receptor-deficient (AG129) mice were used for the prime-boost immunization studies since AG129 mice allow for the efficient replication of LAV for dengue vaccine testing 38,39. The time interval between the two-dose prime-boost immunizations was 3 weeks, and sera were collected 2 weeks after the booster immunizations (**Fig. 2A**). Serum IgG titers against four DENV serotypes were determined by ELISA coated with recombinant DIII proteins or DENVs. The results indicated that the DIII-specific IgG titers for the two-dose FliC-DIII(1-4) and LAV(2,4)+FliC-DIII(2,4) prime-boost immunization groups were higher than the two-dose LAV(2,4) immunizations for the four DENV serotypes (**Fig. 2B**). In contrast, the DENV-specific IgG titers for the two-dose LAV(2,4) group were higher than the other two immunization groups (**Fig. 2C**). For neutralizing antibody titers, the LAV(2,4)+FliC-DIII(2,4) prime- boost immunization group had significantly higher FRNT50 values than those of the two-dose FliC-DIII(1-4) or two-dose LAV(2,4) immunization groups against DENV2 and DENV4, but had similar ranges against DENV1 and DENV3 (**Fig. 2D**). The increase of neutralizing antibody titers did not correlate with the anti-DENV total IgG titers, where the two-dose LAV(2,4) immunization had the highest titers compared to two-dose FliC-DIII(1-4) or the LAV(2,4) + FliC-DIII(1-4) priming boost immunizations.

The model of newborn mice by intracranial challenges was used to evaluate the passive protection elicited by prime-boost immunizations against DENV infections as it has been used to evaluate vaccine formulations 40-42. In the study, the antisera from each of the immunized groups were pooled and mixed with 10^4^ FFU DENV1, DENV2, DENV3, or DENV4, and then transferred to 1-day-old newborn 129 mice for passive protection. The results showed that the group of newborn 129 mice that received LAV(2,4)+FliC-DIII(1-4) antisera had improved protection against DENV1, DENV2, DENV3, and DENV4 infections, leading to survival rates of 50%, 100%, 30%, and 80%, respectively (**Fig. 2E**). Comparatively, the antisera from the two-dose LAV(2,4) group provided the newborn mice with the relatively lower levels of survival rates of 30% (DENV1), 50% (DENV2), 0% (DENV3), and 50% (DENV4) (**Fig. 2E**). Almost no or only 20% protection was observed for their survival rates after two-dose FliC-DIII(1-4) immunizations (**Fig. 2E**). The LAV(2,4)+FliC-DIII(1-4) prime-boost immunization group provided the best protection against the four DENV serotypes. Therefore, prime-boost immunizations with tetravalent FliC-DIII(1-4) booster can further recall DIII-specific neutralizing antibodies to improve protection.

Since the potential risk of ADE of DENV infection may be a major limiting factor in DENV vaccine development, we investigated each group of antisera for *in vitro* ADE assays using K562 cells infected with four serotypes of DENVs. The ADE effects are indicated as the dose-dependent increase than decrease of their fold enhancements of virus infection in K562 cells. The results showed that antisera of the prime-boost LAV(2,4)+FliC-DIII(1-4) and the two-dose LAV(2,4) immunized groups resulted in significant infection enhancements for ADE activity against DENV1 and DENV3, but only minor or no ADE activity against DENV2 and DENV4 (**Fig. 2F**). No ADE activity was found for antisera of two-dose FliC-DIII(1-4) group against four-serotype DENVs (**Fig. 2F**). Therefore, prime-boost immunizations with LAV(2,4) and FliC-DIII(1-4) elicited more potent neutralizing antibodies against four-serotype DENVs but still resulted in significant infection enhancements of ADE activity in K562 cells against DENV1 and DENV3.

### DENV or ZIKV adenoviral vectors priming, followed by FliC-DIII booster immunizations in BALB/c mice

**Figure 3:**
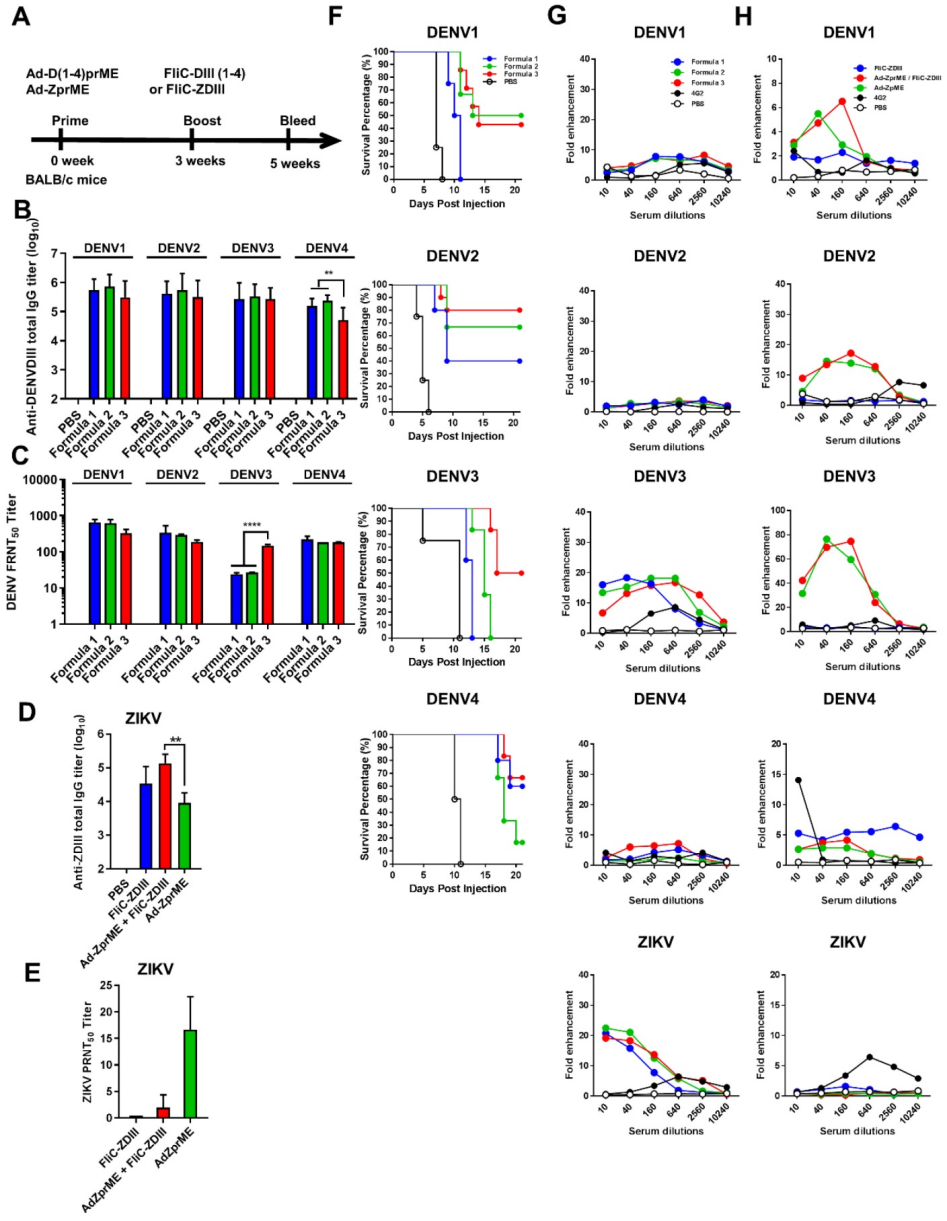
** Adenoviral vectors priming, followed by FliC-DIII booster immunizations in BALB/c mice. (A)** The immunizations were conducted by priming four-serotype DENV (Formulas 1-3) or ZIKV prME-encoding adenovirus vectors, followed by FliC-DIII(1-4) or FliC-ZDIII boosting immunizations in BALB/c mice. PBS-immunized group was the control. Sera were collected from the mice two weeks after the boost immunization. **(B)** Serum total IgG titers against four DENV serotypes of recombinant DIII were measured by ELISA. **(C)** Neutralizing antibody titers against DENV1, DENV2, DENV3, or DENV4.** (D)** Serum total IgG titers against ZIKV DIII were measured by ELISA. **(E)** Neutralizing antibody titers against ZIKV. **(F)** Antisera passive protection in newborn mice. Antisera from the DENV adenovirus vectors priming, followed by FliC-DIII(1-4) booster for Formulas 1-3 groups, and the PBS-immunized control group were mixed with DENVs, and then injected into to newborn BALB/c mice intracranially for measuring antisera passive protection. The survival rates of mice were recorded daily. **(G)** ADE activity of DENV or ZIKV infections mediated by antisera for Formulas 1-3 priming with DENV(1-4) prM-encoding adenovirus vectors, followed by FliC-D1-4(III) booster immunizations. **(H)** ADE activity of DENV infection mediated by priming with ZIKV prME-encoding adenovirus vectors, followed FliC-ZDIII booster immunization, and two doses of FliC-ZDIII or ZIKV prME-encoding adenovirus vectors.The mAb 4G2 was the positive control. The fold enhancement of ADE activity was calculated by dividing the percentage of infected cells with antisera by the percentage of infected cells without antisera. Data were analyzed using one-way ANOVA. (**, p <0.01; ***, p <0.001 ).

To further investigate tetravalent DENV priming and their contributions to elicit serotype-specific neutralizing and cross-reactivity non-neutralizing antibodies, we then switched systems from bivalent DENV LAV (2,4) to adenovirus vectors. We constructed recombinant Ad vectors expressing the full-length prME genes of four-serotype DENVs for tetravalent priming. The prime-boost immunization studies were then conducted by tetravalent DENV Ad vectors priming, followed by tetravalent DENV FliC-DIII boosting. To investigate the prime-boost immunization regimens against the four-serotype DENVs and ZIKV, we constructed recombinant adenovirus vectors expressing the full-length prME gene of four serotypes of DENV (Ad-D1prME, Ad-D2prME, Ad-D3prME, Ad-D4prME) and ZIKV (Ad-ZprME). For the four serotypes of DENV, we investigated three different formulations with different adenovirus titers for priming: Formula 1 (10^8^ PFU Ad-D1 prME, 10^8^ PFU Ad-D2 prME, 10^8^ PFU Ad-D3 prME, 10^8^ PFU Ad-D4 prME), Formula 2 (10^8^ PFU Ad-D1 prME, 10^8^ PFU Ad-D2 prME, 5 x 10^8^ PFU Ad-D3 prME, 10^8^ PFU Ad-D4 prME), and Formula 3 (5 x 10^8^ PFU Ad-D1 prME, 10^8^ PFU Ad-D2 prME, 10^9^ PFU Ad-D3 prME, 10^8^ PFU Ad-D4 prME), and boosting with FliC-D1DIII (20 μg)+ FliC-D2DIII (20 μg)+ FliC-D3DIII (20 μg)+ FliC-D4DIII (20 μg). The immunizations were conducted by priming four- serotype DENV prME-encoding adenovirus vectors, followed by tetravalent FliC-DIII(1-4) booster immunizations in BALB/c mice (**Fig. 3A**). The results indicated that all three formulas elicited similar levels of total serum anti-DENV DIII IgG titers against DENV1, DENV2, DENV3, and DENV4 DIII proteins (**Fig. 3B**). All three formulas had similar FRNT50 values for neutralizing antibodies against DENV1, DENV2, and DENV4, but only Formula 3 had a significantly higher FRNT50 value against DENV3 (**Fig. 3C**). Again, the antisera from each of the immunized groups were pooled and mixed with 10^4^ FFU DENV1, DENV2, DENV3, or DENV4, and then transferred to BALB/c newborn mice for passive protection. The antisera from Formula 1 did not protect against DENV1 and DENV3, but had a 40% survival rate against DENV2 and a 60% survival rate against DENV4 (**Fig. 3F**). The antisera from Formula 2 did not protect against DENV3 but had a survival rate of 50% against DENV1, 70% against DENV2, and 20% against DENV4 (**Fig. 3F**). Mice that received Formula 3 antisera were found to have the highest levels of protection, with survival rates of 40%, 80%, 50%, and 60% against all four serotypes of DENVs (**Fig. 3F**). These data demonstrated that Formula 3 provided the best protection against all four DENV serotypes, in particularly for DENV3, and correlated with the FRNT titers tested in antisera.

For ZIKV, we also investigated the immunizations by Ad-ZprME priming, followed by FliC-ZDIII boosting and compared the results with those of two-dose Ad-ZprME or FliC-ZDIII immunizations. Groups of BALB/c mice were immunized with the following three regimens over a 3-week period: (a) two 20 ug doses of ZIKV FliC-DIII plus Alum (FliC-ZDIII), (b) 10^8^ pfu of adenoviral vectors expressed ZIKV prME primed/20μg of FliC-ZDIII plus Alum boosted (Ad-ZprME+FliC-ZDIII), and (c) two 10^8^-pfu doses of Ad-ZprME. A PBS group was the negative control. The results indicated that the Ad-ZprME+FliC-ZDIII group elicited higher total IgG titers against ZIKV DIII protein than the Ad-ZprME and FliC-ZDIII groups (**Fig. 3D**). However, the neutralizing antibody titers of the Ad-ZprME+FliC-ZDIII and FliC-ZDIII groups were lower than that of the Ad-ZprME group (**Fig. 3E**).

We further investigated the antisera obtained from both DENV and ZIKV adenoviral vector priming and FliC-DIII booster immunizations for their ADE effects on DENV1-4 and ZIKV infected K562 cells. The results showed that the antisera from the three regimens (Formulas 1, 2, and 3) using tetravalent DENV adenoviral vector Ad-D(1-4)prME-primed and the four FliC-DIII(1-4) serotype immunization did not induce ADE effects against DENV1, DENV2, or DENV4 infections (**Fig. 3G**). However, we observed a peak of around 20-fold enhancements of ADE against DENV3 and ZIKV infection (Fig, 5B). Similarly, the antisera from the two-dose Ad-ZprME and the prime-boost Ad-ZprME+FliC-ZDIII groups induced cross-reactive ADE against the four DENV serotypes, particularly for DENV2 and DENV3 infections (**Fig. 3H**).

### Abolishing ADE activity for infection enhancements by glycan-masking E antigens

**Figure 4:**
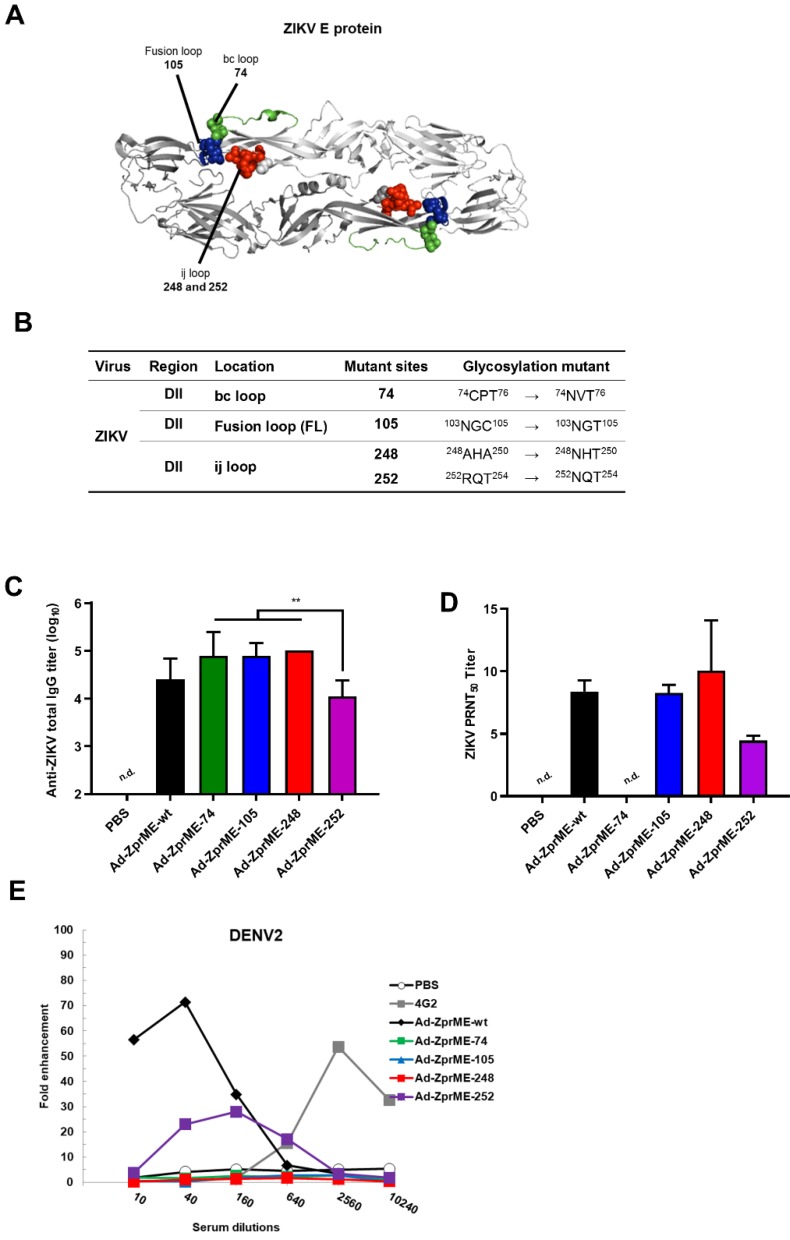
** Reducing ADE activity of infection enhancements by glycan-masking ZIKV E antigen. (A)** Glycan-masking ZIKV E antigen. The structure of ZIKV E protein (PDB: 5JHM) are generated using the PyMOL molecular graphics system (Schrödinger, LLC). Glycan-masking Ad-ZprME mutants by introducing additional N-linked glycosylation motifs on bc loop (green), FL (blue), and ij loop (red). **(B)** A list of four glycan masking mutants constructed by introducing additional N-linked glycosylation motifs on ZIKV E proteins. **(C)** BALB/c mice were immunized with two doses of Ad-ZprME, Ad-ZprME-74, Ad-ZprME-105, Ad-ZprME-248, and Ad-ZprME-252 within a two-week interval, and antisera were collected 2 weeks after second-dose immunization. Serum total IgG titers against recombinant ZDIII were measured using ELISA coated with ZIKV. **(D)** Neutralizing antibody titers against ZIKV were analyzed by PRNT assay and calculated for PRNT_50_ values **(E)** Antisera tested for ADE activity in K562 cells against DENV2 virus infections. The mAb 4G2 was the positive control. The fold enhancement was calculated by dividing the percentage of infected cells with antisera by the percentage of infected cells without antisera. Data were analyzed using one-way ANOVA. (**, p <0.01).

To abolish the production of cross-reactive ADE-facilitating antibodies by ZIKV prime-boost immunizations, we constructed four glycan-masking Ad-ZprME mutants by introducing additional N-linked glycosylation motifs in the DII region of ZIKV E protein (**Fig. 4A**), including the residues ^74^NVT^76^ in the bc loop, the residues ^103^NGT^105^ in the fusion loop, and the residues ^248^NHT^250^ and ^252^NQT^254^ in the ij loop (**Fig. 4B**). Two-dose immunizations by Ad-ZprME, Ad-ZprME-74, Ad-ZprME-105, Ad-ZprME-248, and Ad-ZprME-252 were conducted in BALB/c mice, and the antisera were collected 2 weeks after second-dose immunization. The results indicated that the ZIKV-specific IgG titers elicited by the Ad-ZprME-74, Ad-ZprME-105, and Ad-ZprME-248 groups were significantly higher than those of the Ad-ZprME-252 immunized group (**Fig. 4C**). Neutralizing antibodies as determined by PRNT50 titers were approximately the same for Ad-ZprME, Ad-ZprME-105, and Ad-ZprME-248 groups, and these values are slightly higher than those of the Ad-ZprME-252 group (**Fig. 4D**). No neutralizing antibodies were detected in the antisera of the Ad-ZprME-74 immunized group (**Fig. 4D**). To investigate whether these glycan-masking mutations can abolish the ADE activity, these antisera were tested in K562 cells against DENV2 virus infections. The results indicated that antisera from the Ad-ZprME and Ad-ZprM-74 immunized groups and the 4G2 mAb group showed a peak of 70-, 30-, and 60-fold enhancements against DENV2 infections in K562 cells (**Fig. 4E**). However, antisera from the glycan-masking Ad-ZprME-105 and Ad-ZprME-248 mutations did not induce ADE activity in K562 cells against DENV2 infection (**Fig. 4E**).

### ZIKV and DENV2 adenoviral vectors priming, followed by FliC-DIII booster immunizations using ij loop glycan masking mutations to reduce cross-reactive ADE activity

**Figure 5:**
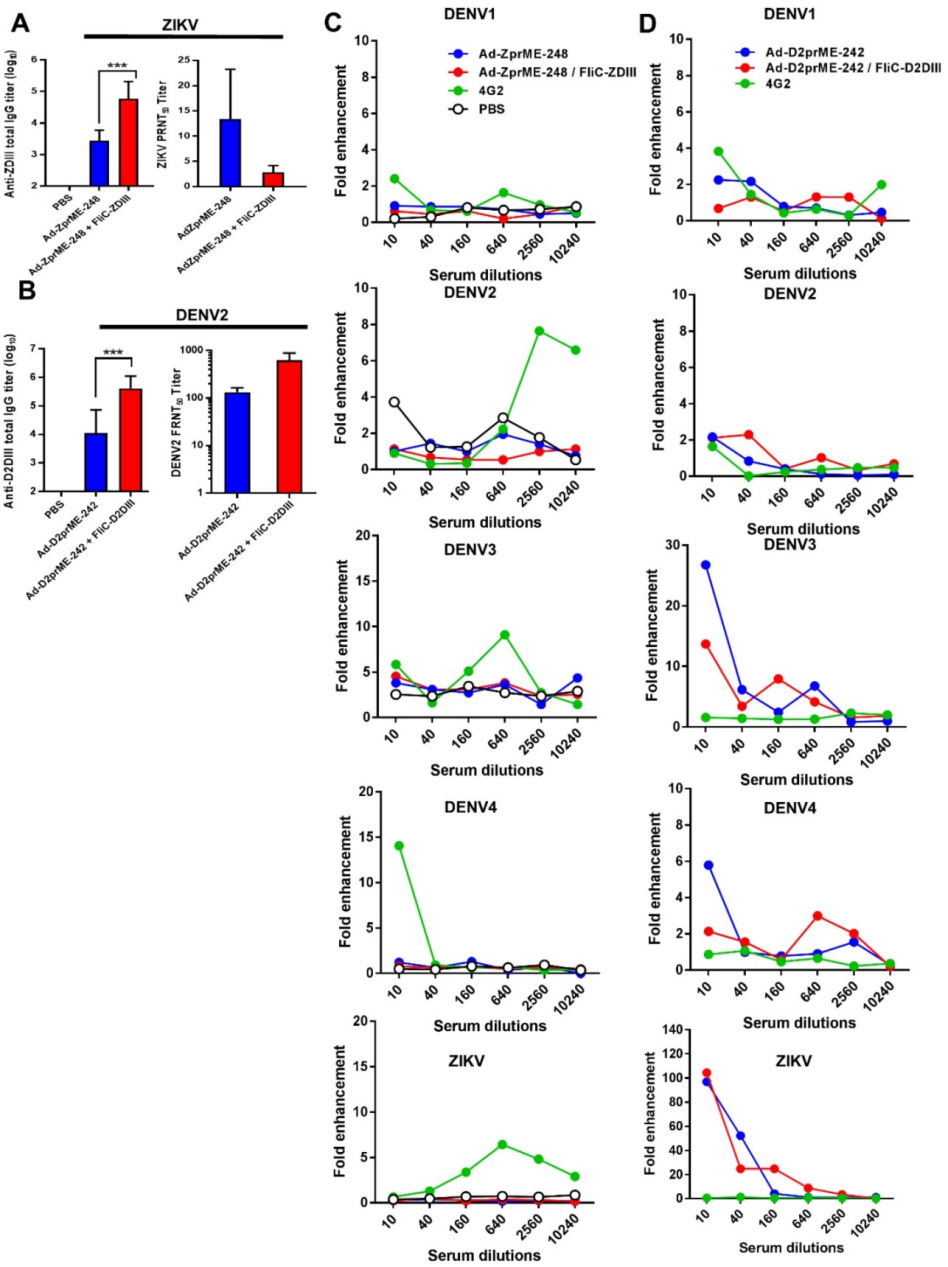
** ZIKV and DENV2 adenoviral vectors priming and FliC-DIII booster immunizations using ij loop glycan masking mutations to minimize cross-reactive ADE effects. (A)** Glycan-masking mutation on the ZIKV E protein ij loop (E-248NHT). Immunizations with two doses of Ad-ZprME-248, the Ad-ZprME-248 priming and FliC-ZDIII booster immunization (Ad-ZprME-248+FliC-ZDIII), and the PBS-immunized control group. Antisera were measured for the titers of ZDIII-specific total IgG and neutralizing antibodies. **(B)** Glycan-masking mutation on the DENV2 E protein ij loop (E-242NHT). Immunizations with two doses of Ad-D2prME-242, the Ad-D2prME-242 priming and FliC-D2DIII booster immunization (Ad-D2prME-242+FliC-D2DIII), and the PBS-immunized control group. Antisera were measured for the titers of D2DIII-specific total IgG and neutralizing antibodies (***, p <0.001). **(C)** ADE activity of DENV or ZIKV infection mediated by Ad-ZprME-248 and Ad-ZprME-248+FliC-ZDIII antisera. **(D)** ADE activity of DENV or ZIKV infection mediated by Ad-D2prME-242 and Ad-D2prME-242+FliC-D2DIII antisera.

Strategies for creating genetic mutations on or near the fusion loops of E protein DII have been previously reported to minimize the cross-reactive ADE effects among different flaviviruses 32-34. As such, we only investigated the ij loop mutation by glycan-masking strategies for prime-boost immunizations. For the ZIKV ij loop, we investigated the Ad-ZprME-248 glycan-masking mutation for adenoviral vectors priming and FliC-DIII booster immunization studies. In parallel, we also constructed a glycan-masking mutation at residue 242 on the DENV2 ij loop and generated the adenoviral vector Ad-D2prME-242, which we then coupled with FliC- D2DIII booster immunization. The results showed that the anti-ZDIII total IgG titer of the Ad-ZprME- 248+FliC-ZDIII group was significantly higher than that of the two-dose Ad-ZprME-248 immunization (**Fig. 5A**). Likewise, antisera from the Ad-D2prME- 242+FliC-D2DIII group showed a higher total IgG titer against D2DIII compared to that from the Ad-D2prME-242 group (**Fig. 5B**). However, the Ad-ZprME-248+FliC-ZDIII prime-boost immunizations did not elicit higher PRNT50 titers than the two-dose Ad-ZprME-248 immunizations for serum ZIKV-neutralizing antibodies (**Fig. 5A**). In contrast, the Ad-D2prME-242+FliC-D2DIII prime-boost immunizations elicited a significantly higher PRNT50 titer against DENV2 compared to the two-dose Ad-D2prME-242 immunizations (**Fig. 5B**). Next, we investigated whether the glycan-masking ij loop mutations could reduce cross-reactivity toward the four serotypes of DENV and ZIKV. The results obtained from *in vitro* ADE assay showed that antisera from the Ad-ZprME-248+FliC-ZDIII prime-boost immunization and the two-dose Ad-ZprME-248 immunizations had the least ADE effects in K562 cells against DENV1, DENV2, DENV3, DENV4, and ZIKV infections as compared to the 4G2 mAb for positive control (**Fig. 5C**). Antisera from the Ad-D2prME-242+ FliC-D2DIII prime-boost immunizations and the two doses of Ad-D2prME-242 immunizations were found to show ADE effects against DENV3 and DENV4, and to a great degree for the peak value for their fold enhancements against ZIKV infections (**Fig. 5D**). These results indicated that only the glycan-masking mutation on the ij loop of ZIKV E DII region (i.e., Ad-ZprME-248) but not on DENV2 E protein ij loop (i.e. Ad-D2prME-242) was able to reduce the cross-reactive ADE activity against infections by the four-serotype DENVs and ZIKV.

## Discussion

Antibody cross-reactivity among the four- serotype DENVs and ZIKV could have significant implications for the development of safe and effective ZIKV vaccines 25. The DENV and ZIKV vaccine design should focus not only on the elicitation of high-titer neutralizing antibodies, but also on the reduction of cross-reactive antibodies leading to disease enhancement. Here, we reported on the use of prime-boost immunization strategies by the second- dose booster of four DENV serotype FliC-DIII proteins, but not ZIKV FliC-DIII proteins, significantly enhanced the neutralizing antibody titers in sera and protection against viral infections. We also constructed the ij loop mutations on ZIKV and DENV2 E antigens using glycan-masking strategies to reduce the elicitation of cross-reactive antibodies for ADE activity. The results indicated that the glycan-masking mutation on the ZIKV E protein ij loop (E-248NHT), but not on the DENV2 E protein ij loop (E-242NHT), resulted in minimizing the production of cross-reactive antibodies for DENV and ZIKV infection enhancements.

To investigate whether the heterologous prime- boost immunization strategy using LAV priming, followed by FliC-DIII booster can elicit more potent neutralizing antibodies, we conducted our experiments using bivalent LAV(2,4) priming, followed by tetravalent FliC-DIII(1-4) boosting immunization in AG129 mice. Compared to the two-dose immunizations by either FliC-DIII(1-4) or LAV(2,4), the LAV(2,4) + FliC-DIII(1-4) prime-boost immunization was found to elicit significantly higher titers of neutralizing antibodies against DENV2 and DENV4, but not against DENV1 and DENV3 infections (**Fig. 2D**). These results of enhanced anti-DENV immunity by prime-boost immunization are consistent with those of a previous report using a monovalent LAV(2) priming, followed by DIII boosting immunizations in monkeys 21. Bacterial flagellin has been identified as one of innate sensors able to trigger TLR5 innate response 43-44. Therefore, flagellin can be used as an adjuvant to enhance antigen immunogenicity 45-46. The present study showed enhanced DIII immunogenicity through FliC fusion in agreement with previous studies using flagellin fusion with the West Nile virus DIII proteins 47 or the DENV DIII proteins 48. Prime-boost immunizations provide an advantage by more effectively recalling DIII antigens to elicit more potent serotype-specific neutralizing antibodies after DIII booster immunizations. Furthermore, we also conducted experiments using bivalent LAV(2,4) priming, followed by either the same serotype bivalent FliC-DIII(2,4) or the different serotype FliC-DIII(1,3) boosting immunization in AG129 mice **(Fig. S1A)**. Homotypic FliC-DIII(2,4) booster immunizations following LAV(2,4) priming were found to elicit high titers of neutralizing antibodies and protection against DENV2 and DENV4, but not DENV1 and DENV3 **(Fig. S1B,D)**. Heterotypic FliC-DIII(1,3) booster immunizations following LAV(2,4) priming resulted in the loss of the ability to elicit neutralizing antibodies and protection against DENV1-3 **(Fig. S1C,E).** These results indicate that LAV priming serotypes were the main determinants for eliciting homotypic but not heterotypic neutralizing antibodies and protection against DENV infections.

An imbalanced immune response with poorly neutralizing cross-reactive antibodies may cause disease enhancement through FcR receptor(s) for ADE effects 23-24. Our results using bivalent LAV(2,4) priming, followed by tetravalent FliC-DIII(1-4) boosting regimens demonstrated ADE effects in K562 cells against DENV1 and DENV3 infections (**Fig. 2F**). Therefore, we constructed four DENV serotypes of adenovirus vectors (Ad-D1prME, Ad-D2prME, Ad-D3prME, Ad-D4prME) and immunized BALB/c mice with three different formulation components. The Formula 3 results were found to elicit high-titer neutralizing antibodies and protection against the four DENV serotypes, in particularly for DENV3 (**Fig. 3C,F**), and no significant ADE effects in K562 cells for DENV1, DENV2, or DENV4 (**Fig. 3G**). The minor enhancement of DENV3 infection may be due to the low neutralizing antibody titer against DENV despite the dose of Ad-D3prME titer in Formula 3 being one-log higher compared to Formula 1. More interestingly, we also observed a peak around 20-fold enhancements of ADE in K562 cells against ZIKV infection (**Fig. 3G**). The ADE effects against the four DENV serotypes, particularly DENV2 and DENV3 infections were also found in sera obtained by immunizations with the ZIKV prME- encoding adenoviral vector (Ad-ZprME) priming, followed either with the same Ad-ZprME or FliC- ZDIII boosting regimens (**Fig. 3H**). The *in vitro* ADE activity and some of the animal model or patient serum results have evidenced the cross reactivity between DENVs and ZIKV 31,49-56, However, the cross-reactivity and disease enhancement for DENV and ZIKV in humans is still unclear. For instance, a recent finding from patients infected with DENV or ZIKV indicated no enhancements of ZIKV viremia or severe diseases compared to naive individuals 57. Another report revealed that DENV-infected patients with ADE activity for severe disease was mainly dependent on the primary antibody titers 58. These primary antibodies in patients have not been extensively studied yet for DENV and ZIKV co-infections.

To abolish the elicitation of cross-reactive ADE-facilitating antibodies, we constructed a series of glycan-masking mutations on DII region of ZIKV E protein (**Fig. 4A-B**). The ij loop is located on the DII of E protein nearby the fusion loop (residues 242-248 of DENV and amino acids 247-253 of ZIKV). In the DENV E:E dimer, the ij loop is in a monomer- monomer contact with a negatively charged region 59. The distance between the ij loop and fusion loop of ZIKV (6.7 Å) is closer than that of DENV2 (12.5 Å). The ij loop is one of the critical region for DENV and ZIKV E-dimer epitope (EDE) mAb binding sites 22,60-63. Our results indicated that the ij loop mutant of ZIKV E antigen was greatly reduced enhancement in all four serotypes of DENV, suggesting that the ij loop mutant is a good target for the glycan-masking strategy to shield the cross-reactive antibodies to induce ADE effects against DENV **(Fig. 4E)**, also retaining the same capability for virus neutralization**(Fig. 4D)**. To our knowledge our findings on the glycan-masking ij loop mutations have never been reported, although other mutations introduced either in or near the FL of DII or the DIII of DENV E proteins have been previously shown to minimize cross-reactivity among different DENV serotypes 32-33. A recent report on ZIKV mRNA vaccine revealed that four mutations (T76R, Q77E, W101R, and L107R) in or near the DII/FL abolished the antibody cross-reactivity of FL-specific antibodies and reduced the ADE effects; however, they still induced lower-titer neutralizing antibodies 34. In the present study, the ZIKV ij loop glycan-masking mutation at residue 248 was found to minimize the cross-reactive ADE effects against DENV or ZIKV infections (**Fig. 5C**), but not the DENV2 ij loop glycan-masking mutation at residue 242 (**Fig. 5D**). Further studies should be conducted to investigate other glycan-masking mutations from the four DENV serotypes to minimize the cross-reactive antibodies for ADE effects against different DENV or ZIKV serotypes.

Finally, we applied prime-boost immunization regimens using the ij loop glycan-masking adenovirus vector, followed by FliC-DIII booster immunizations. Our results indicated only an improvement in the induction of virus-neutralizing antibody titers by tetravalent DENV FliC-DIII boosting immunizations (**Fig. 5B**). However, no enhancing effects eliciting more potent neutralizing antibodies were observed by ZIKV FliC-DIII boosting immunization (**Fig. 5A**). These results will require further investigation since DENV DIII has been shown to be less immunodominant in humans 64-65. The significantly less lower immunogenicity of ZIKV DIII as compared to the four DENVs' DIIIs in mice observed in our studies may reflect for the failure of ZIKV DIII boosting to improve immunogenicity. It has been recently reported that the ZIKV DIII-primed B cell population from all B cells recognizing the ZIKV E protein in patients was only around 6% 66. In summary, our results demonstrated that prime-boost immunization with FliC-DIII boosting can elicit more potent neutralizing antibodies by DENV DIII but not ZIKV DIII antigens. However, glycan-masking shielding on the ij loop of the ZIKV E antigen was successful in minimizing the elicitation of cross-reactive ADE antibodies against DENV infections. These findings could provide useful information for the development of more effective and safe ZIKV and DENV vaccines.

## Methods

### Construction and expression of recombinant proteins

The flagellin (FliC) gene, DIII genes, and FliC-fused DIII were each cloned into the protein expression vector pET22b (+) (Novagen) with a C-terminal His-tag. The FliC gene was derived from *Salmonella typhimurium* (accession No. D13689) and the DIII gene was derived from DENV1, DENV2, DENV3, DENV4, or ZIKV. For the construction of FliC-fused DIIIs, the FliC and DIIIs genes were linked by an amino acid linker of four repeats of glycine and serine (GS_4_ linker). For the expression of the recombinant proteins, *E. coli* BL21 (DE3) (Invitrogen) was transformed with one of the protein expression vectors and cultured at 37°C overnight. The transformed BL21 cells were subcultured inl LB broth and then induced by IPTG. The cells were centrifuged, resuspended in binding buffer, and disrupted by pressure homogenization. The inclusion bodies were collected and resuspended in 8 M urea. The solubilized inclusion bodies were loaded onto a nickel-chelating affinity column comprising Ni-NTA resin (TOSOH), washed with binding buffer with 0.5% Triton X-100, and eluted with elution buffer using the AKTA prime purification system. The purified fractions of the recombinant proteins were concentrated in PBS using an Amicon 3K or 10K filter unit (Merck Millipore) and stored at -20^o^C. Protein samples with SDS-loading buffer were heated and electrophoretically separated by SDS PAGE. Gels were then stained with Coomassie blue solution overnight then destained with destaining solution.

### TLR5-dependent functional assay

293A cells were cultured overnight in a 10-cm culture dish and then co-transfected with 7.5 μg of pUNO1-hTLR5 plasmid (InvivoGen) and 5.5 μg of pGL4.32 [luc2p/NF-κB-RE/Hygro] plasmid (Promega) using TurboFect transfection reagent (Fermentas). The next day, the transfected cells were seeded at a density of 5 × 10^4^ cells/well in a 96-well plate, followed by the addition of a 10-fold serial dilution of the recombinant FliC protein or the recombinant FliC-DIII fusion proteins, which were first diluted to 1 μg/ml in DMEM. After incubating for 5 h, the cells were disrupted using Glo lysis buffer (Promega) and treated with Neolite luciferase substrate (PerkinElmer). After 5 min, a VICTOR3 Multi-labeled Microplate Reader (PerkinElmer) was used to read the 96-well plate for the measurement of the relative luminescence units (RLUs).

### Cells and antibodies

Vero, Vero E6, and BHK21 cells were obtained from the Bioresource Collection and Research Center (BCRC) and cultured in MEM (Gibco) supplemented with 10% heat-inactivated FBS and 100 U/ml of penicillin/streptomycin (P/S). 293A (Invitrogen) and CHO-K1 (BCRC) cells were maintained in DMEM (Gibco) with 10% FBS and 100 U/ml of P/S. Mouse hybridoma cells producing mAbs 2H2 (anti-DENV complex prM), 4G2 (anti-flavivirus envelope FL), and K562 (BCRC) cells were cultured in IMDM (Invitrogen) supplemented with 10% FBS and 100 U/ml of P/S_._ Mammalian cell lines were maintained at 37°C with 5% CO_2_. Mosquito cell line C6/36 cells (BCRC) were grown in Leibovitz-15 medium (Invitrogen) containing 10% FBS, 0.3% tryptose phosphate broth (TPB), 1% non-essential amino acid (NEAA), 25 mM HEPES buffer, and 100 U/ml P/S at 28°C.

### Viruses

DENV1 (Hawaii strain), DENV2 (NGC strain), and DENV3 (H87 strain) were provided by the Center of Disease Control, Department of Health, Taiwan and prepared from supernatants of infected C6/36 cells. ZIKV (PRVABC59 strain) was obtained from the American Type Culture Collection (ATCC) and amplified in Vero cells. The infectious cDNA plasmids of DENV4 containing full-length cDNA copies of DENV4 strain 814669, its 3' noncoding region deletion vaccine candidate strain DENV4Δ30, and DENV2/4Δ30 containing the prM and E genes of the DENV2 NGC strain were obtained from the US NIH. Culture supernatants were collected at 8 days post-transfection. To prepare high DENV titers, viruses were concentrated using a 10-kDa Amicon Ultra-15 Centrifugal Filter (Millipore). All virus stocks were stored at -80^o^C until further used.

### Determination of virus titer

DENV titers were determined by focus-forming- unit (FFU) assay. Vero E6 cells were seeded in 6-well plates at a density of 5 x 10^5^ cells/well. After being left overnight, serial 10-fold dilutions of virus were added, and each dilution was performed at least in duplicate. After incubation at 37^o^C for 1 h, 4 ml of overlay medium containing 1× MEM, 1.1% methylcellulose, and 100 U/ml of P/S was added to each well, and the overlay medium was removed after 7-10 days post-incubation. The infected cells were fixed by 4% formaldehyde for at least 1 h, washed three times with 0.05% Tween 20 in PBS (PBST), and then incubated for 1 h with 40 2H2 mAbs diluted 1:400 in PBS. After another three washes in PBST, the cells were incubated with goat anti-mouse IgG HRP secondary antibody (GeneTex) and diluted 1:1500 in PBS for 1 h. Cells were stained with a Liquid DAB-Plus Substrate Kit (Invitrogen) after being washed three times with PBST for 20 min. The number of DENV-infected cells was visibly quantified and reported as FFU. ZIKV titers were determined by plaque assay. Vero cells were seeded in 6-well plates at a density of 5 x 10^5^ cells/well. After being left overnight, serial 10-fold dilutions of virus were added, and each dilution was performed in at least duplicate. After incubation at 37^o^C for 1 h, 4 ml of overlay medium containing 1× MEM, 1.1% methylcellulose, and 100 U/ml of P/S was added to each well, and the overlay medium was removed after 5 days post-incubation. Virus plaques were fixed and stained with 1% crystal violet and 2% formaldehyde for 2 h. Visible plaques were counted and the virus titers (PFU) were calculated.

### Preparation of adenoviral vectors expressing DENV and ZIKV prME

Adenoviral vectors expressed prM protein and E protein (both proteins collectively referred to as prME) with the signal peptide (20 amino acids at the 3' terminal of C protein) of DENV1, DENV2, DENV3, DENV4, or ZIKV. The cDNA of viruses containing the prME gene fragment was used as the source of prME. The DENV3, DENV4, or ZIKV prME with signal peptide gene sequences were optimized for human codon usage and generated by gene synthesis (Genomics). Each of the prME gene fragments was PCR amplified using different primer sets, inserted into the transfer vector pENTR1A (Invitrogen) separately, and then cloned into the adenoviral plasmid pAd/CMV/V5-DEST (Invitrogen) using LR ClonaseTM II Enzyme Mix (Invitrogen) to generate the adenoviral plasmid expressing prM and E proteins. To obtain adenovirus particles, the adenoviral plasmids were cleaved with Pac I restriction enzyme to expose the inverted terminal repeats (ITRs) and then transfected into 293A cells separately using TurboFect transfection reagent (Fermentas). After 10-15 days, the transfected cells and culture media were collected once the cytopathic effects were visible. The cells were disrupted by freeze-thaw cycles three times to release the intracellular viral particles, and the supernatants of the cell lysates were collected by centrifugation (3000 rpm, 15 min, 4^o^C) to obtain the adenoviral vectors expressing the prM and E proteins of each virus, referred to as Ad-D1prME, Ad-D2prME, Ad-D3 prME, Ad-D4prME, and Ad-ZprME. To prepare higher titers, the virus was concentrated using a 30-kDa Amicon Ultra-15 Centrifugal Filter (Millipore). The viral stocks were stored at -80^o^C. To determine the adenovirus titers, 293A cells were seeded in 6-well plates at a density of 10^6^ cells/well and incubated at 37^o^C overnight. The 10-fold serially diluted adenoviruses were then added to each well for 24 h at 37^o^C. Next, the media containing the diluted adenoviruses were removed, and 3 ml/well of DMEM medium containing 0.4% agarose and 100 U/ml P/S was added to the 6-well plates. The plaques were visibly quantified 7-10 days after the cells were infected with adenoviruses and the PFU was reported.

### Mouse immunizations

BALB/c and 129 (National Laboratory Animal Center, Taiwan) or AG129 (purchased from B&K Universal)) mice aged 6-8 weeks were used for vaccination since DENV LAV was able to replicate in AG129 mice (which lack IFN-α/β and IFN-γ receptor genes). The mice were immunized with prime-boost regimens at weeks 0 and 3. The immunization regimens containing an LAV (10^4^ FFU per dose) or an adenoviral vector (10^8^ PFU per dose) were administered via intraperitoneal injection. The immunizations containing recombinant FliC-DIII proteins (20μg per dose for each subtype) of DENV were administered via intramuscular injection. The immunizations containing recombinant FliC-ZDIII proteins (20μg per dose) were formulated with alum and administered via intramuscular injection. All vaccines were prepared as a dosage using PBS as the diluent in a final volume of 200 μl. Mice were bled on week 5, and the blood samples were centrifuged at 3000 rpm for 15 min to isolate the serum from the blood cells. The sera were inactivated by heating at 56^o^C for 30 min and stored at -20^o^C. All procedures involving animals were performed in accordance with guidelines established by the Laboratory Animal Center of National Tsing Hua University (NTHU). Animal use protocols were reviewed and approved by the NTHU Institutional Animal Care and Use Committee (approval no. 10246).

### Enzyme-linked immunosorbent assay (ELISA)

The total serum IgG titers against the recombinant DIII proteins or virions were determined by ELISA. The ELISA plate was coated with 0.2 μg of the recombinant DIII proteins or 10^5^ FFU formalin-inactivated virus (100 μl/well) in 0.05M carbonate buffer at 4^o^C overnight. After being washed with PBST three times, each well of the plate was blocked with 150 μl/well of 1% bovine serum albumin (BSA) in PBS for 1 h at room temperature (RT) and then washed with 200 μl PBST three times. Next, the mouse sera were 4-fold serially diluted from a dilution factor of 1:100 with dilution buffer (PBS containing 1% BSA and 0.05% Tween 20) and added to the ELISA plate for 1 h of incubation at RT. After washing with PBST three times, each well of the ELISA plate was treated with 100 μl HRP-conjugated goat anti-mouse IgG antibody at a dilution factor of 1:10000 at RT for 1 h and washed with PBST three times. Finally, 100 μl/well of 3,3′,5,5′-tetramethylbenzidine (TMB) substrate (BioLegend) was added to the ELISA plate for coloration and allowed to react in the dark for 15 min. The reaction was stopped by 100 μl/well of 2 N sulfuric acid. The absorbance for each well was measured at 450 nm using an ELISA reader (DYNEX MRX II). The end-point titers of total IgG were defined as the maximal serum dilutions that produced an OD value of over 0.2.

### Virus neutralization assay

The serum neutralization against DENV was determined by a focus-reduction neutralization test (FRNT). The antisera were 2-fold serially diluted from a dilution factor of 1:4 in Hank's balanced salt solution (HBSS) containing 0.4% BSA fraction V and incubated with 200 FFU DENV1, DENV2, DENV3, or DENV4 at 37^o^C for 1 h. Next, the sera-virus mixtures were added to 6-well plates seeded with monolayer Vero E6 cells for the determination of FFU by FFU assay. The plaque-reduction neutralization test (PRNT) was used for measuring neutralizing antibodies against ZIKV. The procedures were the same but instead for the determination of PFU by plaque assay. A reduction in focus or plaque number was used to calculate the neutralization percentage and plot the neutralization curves. The neutralizing antibody titer, represented by FRNT_50_ or PRNT_50_, was defined as the maximal serum dilution that caused a reduction of over 50% in focus or plaque number and was determined by regression analysis using GraphPad Prism version 6.

### Antisera passive protection in newborn mice

The use of newborn intracranial challenges was used to evaluate the passive protections elicited by prime-boost immunizations against DENV infections as it has been used to evaluate vaccine formulations 40-42. Mouse sera were incubated with 10^4^ FFU DENV1, DENV2, DENV3, or DENV4 at a ratio of 1:1 to a final volume of 30 μl at 37^o^C for 1 h. One-day-old newborn 129 or BALB/c mice were inoculated with the sera-virus mixtures by intracranial injection. After inoculations, infected newborn mice were observed for developing disease signs like bristling hair, lordosis, paralysis and finally death between 7 to 10 days, and recorded on a daily basis for the mortality and morbidity rates during 21 days after infection.

### ADE assay

Four-fold serial dilutions from a dilution factor of 1:10 of mouse sera with IMDM were incubated with DENV1, DENV2, DENV3, DENV4, or ZIKV at an MOI of 1 at 37^o^C for 1 h. The mixtures were the incubated with 2 x 10^5^ K562 cells at 37^o^C for 2 h. After infection, the cells were washed twice with IMDM and seeded in 24-well plates with IMDM at 37^o^C for 2 days. The infected cells were washed with PBS twice (centrifuged at 500 g, 5 min, 4^o^C), fixed with 4% formaldehyde at RT for 30 min or at 4°C overnight, and permeabilized in PBS containing 1% BSA and 0.5% saponin (Sigma) at RT for 20 min. After being washed with FACS buffer (PBS containing 1% BSA and 0.1% saponin) twice, the cells were incubated with mAb 4G2 conjugated to AlexaFluor-488 (Invitrogen) at a dilution factor of 1:200 in FACS buffer at RT for 30 min. After being washed with FACS buffer twice, the cells were resuspended in PBS and analyzed using a BD Accuri™ C6 Cytometer with BD Accuri C6 Software (BD Biosciences). Fold enhancement was defined as the percentage of infected cells with sera or mAb divided by the corresponding percentage of infected cells without sera or mAb.

### Statistical analysis

Statistical analyses were performed using GraphPad Prism (GraphPad Software, Inc.). The statistical significance of the differences between groups was assessed using one-way ANOVAs. Differences with a *P*-value of less than 0.05 (*), 0.01 (**), 0.001 (***) were considered statistically significant. All experiments were repeated at least twice.

## Supplementary Material

Supplementary figure.
