# Supplementary material for: Dengue and Zika Virus Domain III-Flagellin Fusion and Glycan-Masking E Antigen for Prime-Boost Immunization
Source: Theranostics. 2019 Jul 9;9(16):4811–26. doi: 10.7150/thno.35919 (PMC6643441; doi:10.7150/thno.35919)
Supplement: Supplementary file 1 — Supplementary figure. [file thnov09p4811s1.pdf]

Dengue and Zika virus domain III-flagellin fusion and glycan-masking E antigen for prime-boost immunization

Hsiao-Han Lin, Shao-Ping Yang, Meng-Ju Tsai, Guan-Cheng Lin, Han-Chung Wu, Suh-Chin Wu

Fig. S1. (A) Prime-boost immunization regimens were conducted using the NIH live-attenuated bivalent vaccine strains (DENV2/4Δ30 and DENV4Δ30, or LAV(2,4)) and bivalent FliC-DIII boosting. Groups of AG129 mice were immunized using LAV(2,4) priming, followed by by either the homotypic bivalent FliC-DIII(2,4) or the heterotypic FliC-DIII(1,3) boosting, two doses of bivalent FliC-DIII(2,4)/FliC-DIII(1,3), or two doses of LAV(2,4). Sera were collected from the mice two weeks after the second dose immunization. **(B)** Neutralizing antibody titers against DENV1, DENV2, DENV3, or DENV4 with the homotypic bivalent FliC-DIII(2,4) boosting. (**C)** Neutralizing antibody titers against DENV1, DENV2, DENV3, or DENV4 with the heterotypic bivalent FliC-DIII(1,3) boosting. **(D)** Passive protection in newborn mice by antisera from the homotypic bivalent FliC-DIII(2,4) boosting. The survival rates of mice were recorded daily.. **(E)** Antisera passive protection in newborn mice. **(F)** A Passive protection in newborn mice by antisera from the heterotypic bivalent FliC-DIII(1,3) boosting. The survival rates of mice were recorded daily. Data were analyzed using one-way ANOVA. (*, $p < 0.05$; ***, $p < 0.001$).

Fig. S1

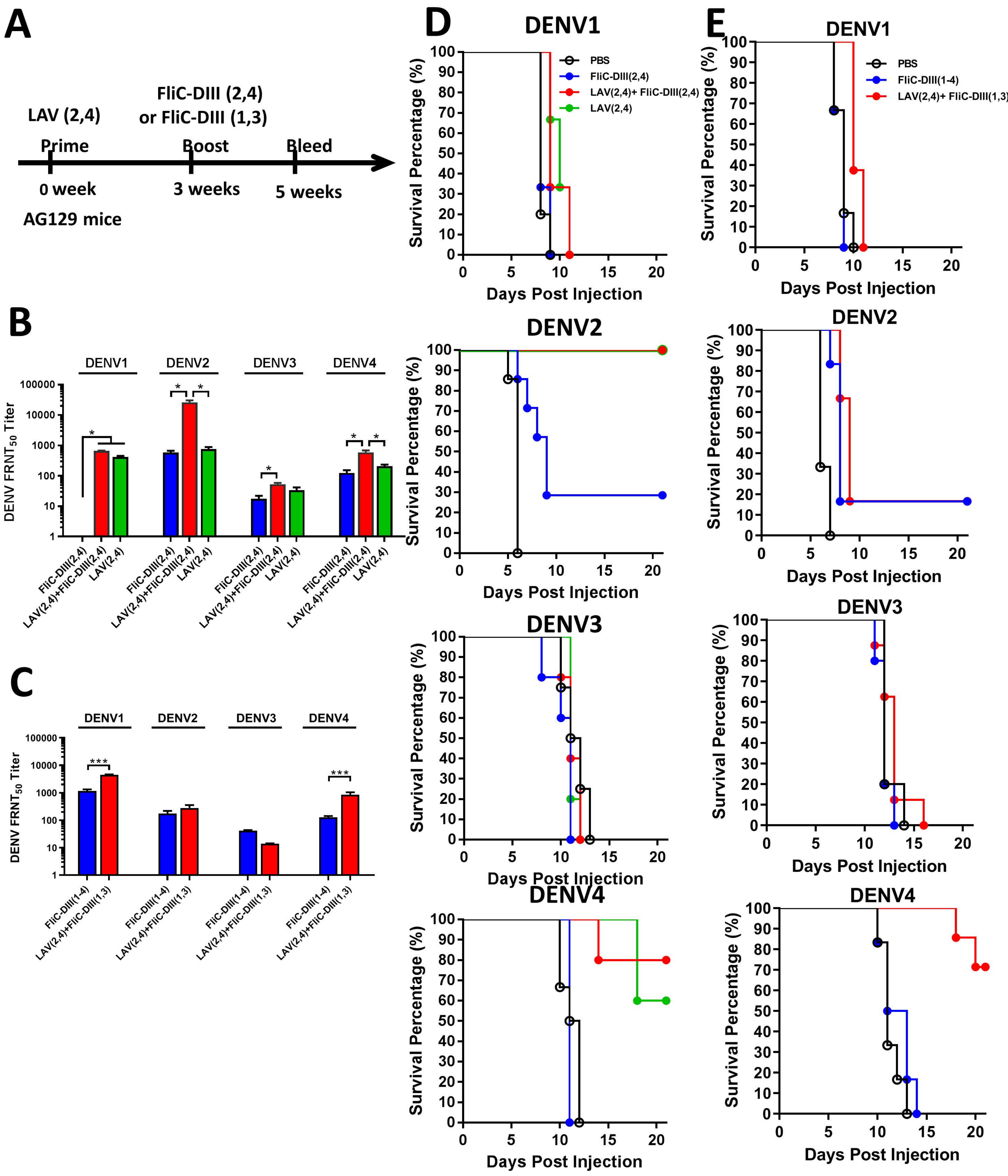
A
LAV (2,4)
FliC-DIII (2,4)
or FliC-DIII (1,3)
Prime
Boost
Bleed
0 week
3 weeks
5 weeks
AG129 mice
B
DENV1
DENV2
DENV3
DENV4
DENV FRNT50 Titer
FliC-DIII(2,4)
LAV(2,4)+FliC-DIII(2,4)
LAV(2,4)
C
FliC-DIII(1-4)
LAV(2,4)+FliC-DIII(1,3)
D
E
PBS
FliC-DIII(2,4)
LAV(2,4)+ FliC-DIII(2,4)
LAV(2,4)
FliC-DIII(1-4)
LAV(2,4)+ FliC-DIII(1,3)
Survival Percentage (%)
Days Post Injection
